# Supplementary material for: Intermediates of forming transition metal dichalcogenide heterostructures revealed by machine learning simulations
Source: Nat Commun. 2026 Feb 23;17:3086. doi: 10.1038/s41467-026-69977-x (PMC13039184; doi:10.1038/s41467-026-69977-x)
Supplement: Supplementary file 1 — Supplementary Information [file 41467_2026_69977_MOESM1_ESM.pdf]

Supplementary Information for

Intermediates of Forming Transition Metal Dichalcogenide Heterostructures Revealed by Machine Learning Simulations

Luneng Zhao, Hongsheng Liu, Yuan Chang, Xiaoran Shi, Jijun Zhao, Feng Ding and Junfeng Gao*

Corresponding authors: Junfeng Gao, gaojf@dlut.edu.cn

The supplementary materials provide further details to substantiate the findings presented in the main manuscript. We include assessments of the precision of the machine learning potential (MLP) developed for our simulations, extended discussions on the unique intermediate structures identified during transition metal dichalcogenide (TMD) growth, and additional simulation results exploring the kinetics of metal atom embedding and exchange. Lastly, we discuss potential applications of the SMMS intermediate structure in electronic devices.

1. Validation of MLP Accuracy

In this study, we employed an iterative training approach to develop the MLP. The process began with the generation of an initial dataset using ab initio molecular dynamics (AIMD) simulations. Subsequently, we assessed the model's reliability by benchmarking it against DFT calculations and applying it to simulate the two-step growth of MoS_2/WS_2. If the MLP exhibited instabilities during molecular dynamics or failed to reproduce accurate phonon spectra, we refined the model by incorporating additional relevant DFT configurations into the training set. This cycle of data expansion and model retraining was repeated iteratively until the MLP achieved satisfactory accuracy and stability. The final optimized model was then deployed for efficient and accurate large-scale atomistic simulations.

To generate the initial training set, we employed on-the-fly molecular dynamics (MD) simulations, a method that effectively explores unknown configuration spaces while minimizing sampling costs. These simulations were conducted using the VASP on-the-fly MD framework with parameters optimized for efficiency and accuracy. Specifically, we utilized a timestep of 3.5 fs to capture atomic motions precisely, with a maximum of 20,000 steps per trajectory. The simulations were performed in the canonical (NVT) ensemble using Nosé-Hoover thermostats, covering a temperature range from 500 K to 1300 K to encompass typical growth conditions. The unit-cell shapes were kept fixed, and the number of local reference configurations in the on-the-fly MLP

was constrained by setting ML_MB=2000. The initial configurations included monolayer MoS_2 and WS_2, as well as their combinations featuring homogeneous/heterogeneous junctions and adsorbed metal clusters. Subsequently, the training set was expanded through iterative training rounds employing distinct sampling strategies:

• **Training round 1:** On-the-fly MD sampling of MSMS structure dynamics.

• **Training round 2:** Randomized metal-sulfur exchange sampling — Starting from the relaxed MSMS structure, we performed random substitutions between metal (Mo/W) and sulfur atoms at specific interfacial sites to generate intermediate SMMS-like configurations. These exchanges emulate early-stage metal–chalcogen mixing observed during the deposition process.

• **Training round 3:** Randomized metal-metal swapping — To construct a diverse set of alloyed SMMS structures, we performed layer-constrained Mo-W swaps across top and bottom layers. This preserves the overall composition but introduces configurational disorder relevant to alloying behaviour.

• **Training round 4:** Surface metal-sulfur exchange sampling — Starting from SMMS structures, we randomly exchanged surface S atoms with underlying metal atoms (Mo or W) to simulate possible sulfurization pathways and local rearrangements during the growth process.

• **Training round 5:** Perturbed configurations near SMMS equilibrium states.

• **Training round 6:** On-the-fly MD sampling of sulfur molecular dynamics (matching initial dataset generation method).

• **Training round 7:** S-S/Mo-S/Mo-W dimer configurations at varied distances.

• **Training rounds 8-17:** MLP-MD simulations of SMMS growth and Mo deposition (key structures extracted every 10,000 steps).

During each training round, we fine-tune the MLP model through 1:1 random sampling of historical data (cumulative from prior training rounds) and newly generated data. The MLP fine-tuning employed a learning rate of 1×10^{-4} to balance convergence speed and accuracy. The final

dataset contained approximately 26,000 configurations from initial training and all training rounds, ensuring structural diversity and representativeness critical for robust MLP development. The dataset has been uploaded to the **Zenodo repository (https://doi.org/10.5281/zenodo.18397127)**.

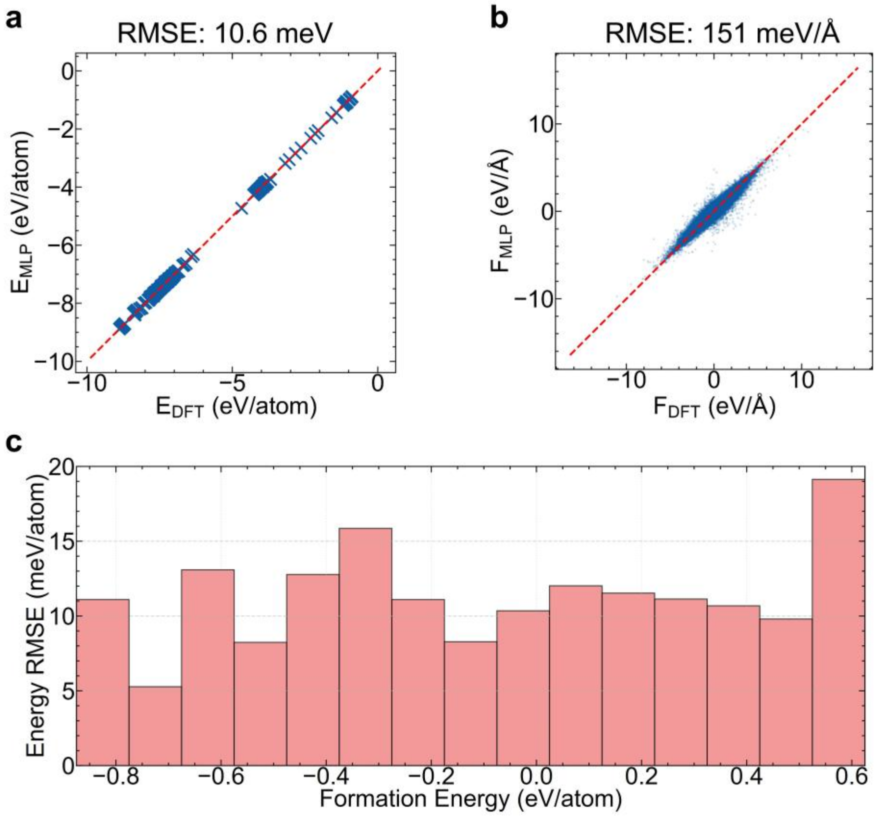

FIG. S1: Comparison of MLP predictions with DFT calculations. (a) Correlation between MLP-predicted energies and DFT-calculated energies per atom. The root mean square error (RMSE) is 10.6 meV/atom. (b) Correlation between MLP-predicted forces and DFT-calculated forces. The RMSE is 151 meV/Å. The red dashed lines represent perfect correlation. (c) Distribution of energy RMSE for test set structures across varying formation energy intervals. The histograms display the prediction errors for energy in meV/atom. The formation energy per atom determines the binning interval.

To assess the MLP's accuracy and reliability for simulating TMD heterostructure growth, we reserved 10% of the overall dataset as a test set. FIG. S1 presents a comparison between MLP

predictions and DFT calculations. As shown in FIG. S1(a), it demonstrates the MLP's precision in predicting atomic energies, with a RMSE of 10.6 meV/atom [1]. FIG. S1(b) demonstrates the MLP's precision in predicting atomic forces, with a RMSE of 151 meV/Å. FIG. S1(c) provides a more detailed error analysis by categorizing the test structures based on their formation energy per atom. The histogram displays the energy RMSE distribution across different formation energy intervals. This analysis reveals that the model maintains exceptional accuracy for structures in the lower energy regimes (typically corresponding to equilibrium or near-equilibrium configurations). Crucially, even for higher-energy states, which represent distorted configurations or transition states frequently encountered during high-temperature growth simulations, the energy prediction errors remain low and bounded. This comprehensive validation confirms the MLP's robustness and consistent accuracy across the entire relevant energy landscape.

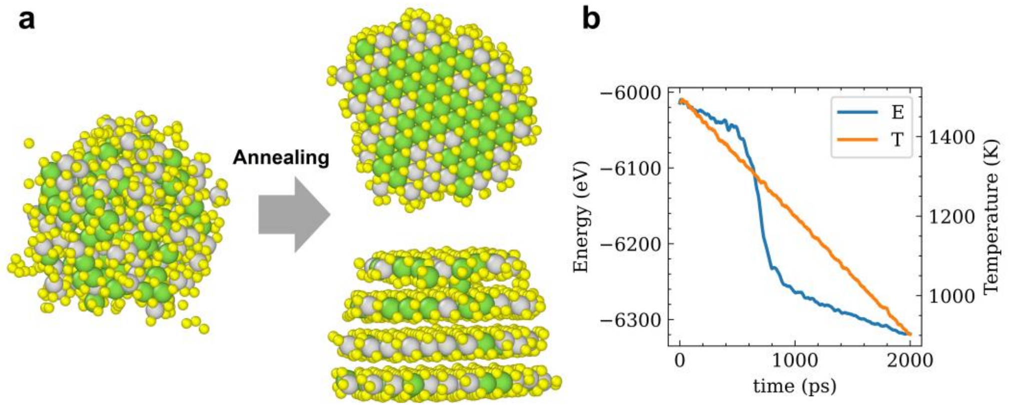

FIG. S2: Formation of ordered TMD layers through annealing simulation. (a) Snapshots depicting the evolution from an initial random mixture of Mo, W, and S atoms (left, ratio 1:1:4) to ordered TMD layers after annealing. (b) Time evolution of system energy (E, blue line) and temperature (T, orange line) during the 2 ns annealing process, illustrating the formation of stable TMD structures. Note: The data has been smoothed using a low-pass filter for clarity.

To validate the MLP's capability in describing growth processes, we conducted simulated annealing experiments. FIG. S2 illustrates the results of these simulations. As shown in FIG. S2(a),

we started with a random mixture of Mo, W, and S atoms (left image) and observed their evolution into ordered TMD layers after annealing (right images, showing top and side views). This transformation demonstrates the MLP's ability to accurately capture the self-assembly process of TMD structures.

FIG. S2(b) presents the evolution of the system energy (E, blue line) and temperature (T, orange line) during the 2 ns annealing process. The decreasing energy trend, coupled with temperature fluctuations, indicates the formation of stable TMD structures. It's worth noting that a low-pass filter was applied to smooth the results, enhancing the clarity of the overall trends. These annealing simulations validate our MLP's effectiveness in modelling TMD growth.

Table S1. Comparison of energies (eV/atom) for critical structures calculated by DFT and MLP

Structure	DFT (eV/atom)	MLP (eV/atom)	ΔE (eV/atom)
S solid	-4.323	-4.311	-0.013
1H-MoS_2	-7.582	-7.583	0.001
1T-MoS_2	-7.298	-7.300	0.002
1T'-MoS_2	-7.390	-7.380	-0.010
1H-WS_2	-8.214	-8.144	-0.070
1T-WS_2	-7.893	-7.899	0.005
1T'-WS_2	-8.026	-8.017	-0.009
MoSWS	-8.375	-8.416	0.041
MoSMoS	-7.978	-8.007	0.029
SMoMoS	-8.409	-8.382	-0.027
SMoWS	-8.895	-8.892	-0.002
SMMS	-8.897	-8.895	-0.002
SMoMoS (from MD)	-8.329	-8.314	-0.015
SMoWS (from MD)	-8.804	-8.815	0.011

Table S2: Dataset statistics (including cell volumes)

Structure Category	Configurations	Avg. Atom Count	Typical Cell Size (Å)
TMD surface with W-Mo-S cluster interaction	18396	155	22 × 22 × 30
Single-layer Mo on WS_2 surface	668	196	22 × 22 × 21
Disordered alloyed TMD structures	1231	144	22 × 22 × 23
1T-phase TMD	1804	144	22 × 22 × 23
Further sulfurized structures of SMMS	360	280	21 × 21 × 21
TMD nanotubes	403	62	32 × 32 × 5
Disordered SMMS structures	900	196	22 × 22 × 21
Ordered SMMS structures	748	144	18 × 16 × 30
MoSWS structures	96	143	19 × 16 × 22
Pristine mono-/bilayer MoS_2/WS_2 and heterostructures	138	58	13 × 10 × 26
Elemental sulfur	1101	35	14 × 14 × 19
Dimers	589	2	25 × 25 × 25
Total	26434		

Table S3: Summary of simulation parameters used in each figure

Figure Reference	Temperature	Ensemble
Fig. 2(a-c)	1100 K	NVT
Fig. 2(d-e)	900 K	NVT
Fig. 3(a-f)	900 K	NVT
Fig. 4	1100 K	NVT
Fig. S4	900 K	NVT
Fig. S6	1100 K	NVT
Fig. S7(a)	1100 K	NVE
Fig. S7(b-c)	1100 K	NVT
Fig. S9	1100 K	NVT
Fig. S10	300 K	NVT
Fig. S11	1100 K	NVT
Fig. S13	1100 K	NVT
Fig. S14	1100 K	NVT
Fig. S15	900 K	NVT
Fig. S16	900 K	NVT
Fig. S17	1100 K	NVT
Fig. S18	1100 K	NVT

2. Formation of SMoMoS Intermediate Structures

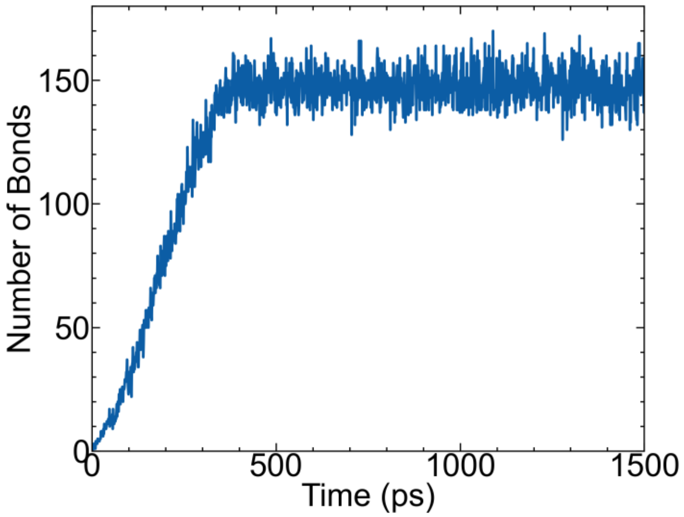

FIG. S3: Number of Mo-Mo bonds during Mo deposition on a MoS_2 substrate. Bond identification is based on a cutoff distance of 2.85 Å, which is larger than the Mo-Mo bond length in Mo bulk (~2.74 Å) but smaller than the equilibrium Mo–Mo spacing in pristine MoS_2 (~3.15 Å).

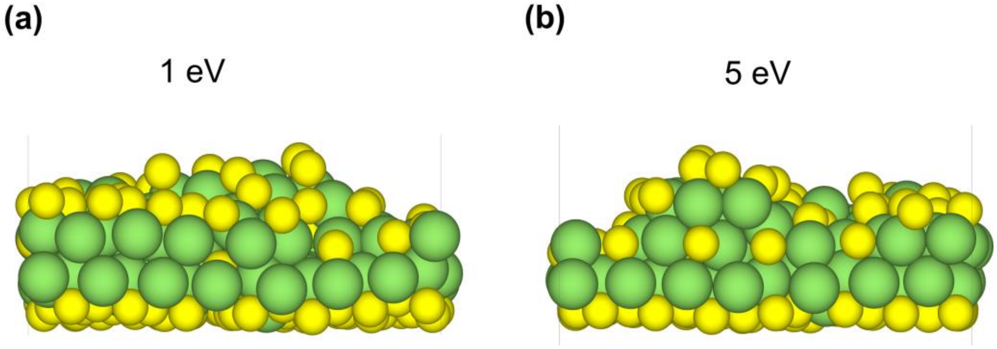

FIG. S4: (a) Simulated 1 eV kinetic energy deposition of a single Mo layer at 900 K on MoS_2. (b) Simulated 5 eV kinetic energy deposition of a single Mo layer at 900 K on MoS_2.

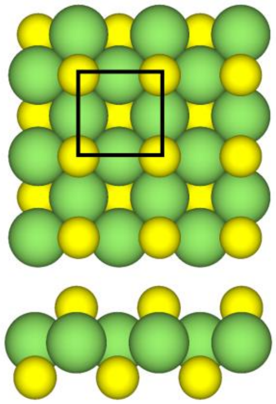

FIG. S5: Structure from MatHub-2d (id: MatHub2d-1817-Mo_2S_2).

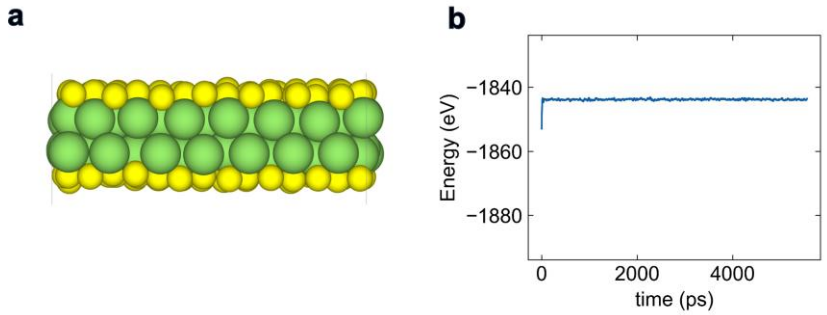

FIG. S6: Stability analysis of SMMS intermediate structures. (a) Atomic configurations of SMoMoS. (b) Corresponding energy evolution during 5500ps MLP-MD simulations at 1100 K for SMoMoS, demonstrating its long-term stability. Note: The data has been smoothed using a low-pass filter for clarity.

3. Validation of the formation of SMoMoS

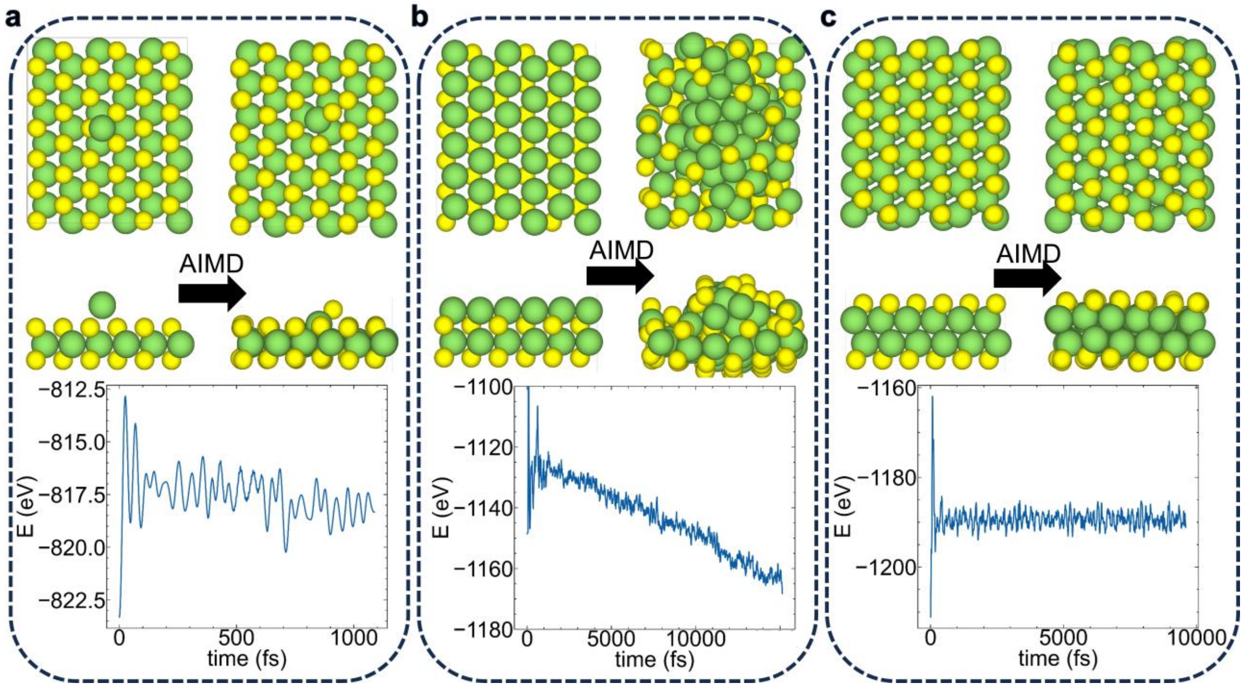

FIG. S7: AIMD simulations validating SMoMoS formation and stability at 1100 K. (a) Single Mo atom embedding into MoS_2 (~1000 fs). Top: Initial and final configurations. Bottom: Energy-time curve showing abrupt decrease at ~700 fs (NVE). (b) Mo atom layer embedding into MoS_2(~15 ps). Top: Initial and final states. Bottom: Energy-time curve during embedding process (NVT). (c) SMoMoS structure stability (~10 ps). Top: Initial and final states. Bottom: Energy-time curve demonstrating structural stability (NVT).

FIG. S7 presents the results of our ab initio molecular dynamics (AIMD) simulations conducted at 1100 K to validate the formation and stability of the SMoMoS structure. These simulations provide crucial insights into the behaviour of Mo atoms on MoS_2 surfaces and the formation mechanism of the SMoMoS intermediate structure.

FIG. S7(a) illustrates the embedding process of a single Mo atom into a MoS_2 layer. We initially positioned a Mo atom 2.3 Å above the MoS_2 surface and imparted it with a downward velocity of 5 Å/ps, corresponding to a kinetic energy of approximately 0.12 eV. This energy is comparable to the thermal energy of atoms at 1100 K. To accurately simulate thermal conditions, we sampled the initial velocities of all atoms from a Boltzmann distribution at 1100 K. The simulation was

performed using the NVE (constant number of particles, volume, and energy) ensemble to capture the system's natural evolution without external temperature control. The top images show the initial and final atomic configurations, while the bottom graph depicts the energy evolution over time. Despite the relatively low initial kinetic energy, the Mo atom rapidly embeds itself into the MoS_2 layer within approximately 700 fs, as evidenced by the abrupt energy decrease. This embedding process releases about 1.45 eV of energy (validated by DFT calculations), demonstrating a strong thermodynamic driving force for Mo incorporation into the MoS_2 structure.

FIG. S7(b) extends our investigation to the behaviour of a layer of Mo atoms on MoS_2. The simulation runs for approximately 15 ps, allowing us to observe the long-term evolution of the system. The top images show the initial configuration with a layer of Mo atoms on the MoS_2 surface, and the final state where multiple Mo atoms have embedded into the MoS_2 layer. The bottom graph illustrates the gradual decrease in system energy as Mo atoms progressively embed into the MoS_2 structure. This energy reduction, significantly lower than the initial configuration, further confirms the thermodynamic favourability of Mo incorporation.

FIG. S7(c) focuses on the stability of the formed SMoMoS structure over an extended period of about 1 ps. The top images demonstrate that the SMoMoS structure remains intact throughout the simulation, with no significant structural changes observed. The bottom graph shows a stable energy curve over time, confirming the structural integrity and thermodynamic stability of the SMoMoS intermediate.

These AIMD simulations serve as a robust validation of our MLP results, confirming the accuracy and reliability of our MLP in predicting the behaviour of Mo atoms on MoS_2 surfaces.

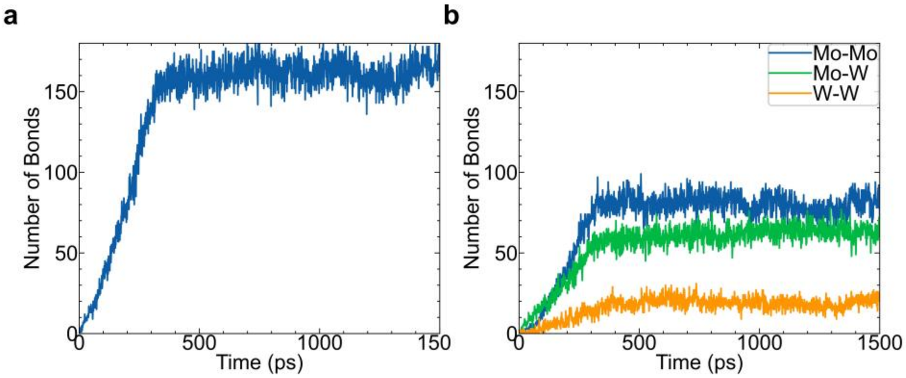

FIG. S8: (a) Evolution of the total number of Mo/W–Mo/W chemical bonds (including Mo-Mo, Mo-W, and W-W bonds) during Mo deposition on a WS_2 substrate. (b) Individual counts of Mo-Mo, Mo-W, and W-W bonds. Bond identification is based on a cutoff distance of 2.85 Å, which is larger than the Mo-Mo bond length in Mo bulk (~2.74 Å) and W bulk (~2.75 Å) but smaller than the equilibrium Mo-Mo spacing in pristine MoS_2 (~3.15 Å)

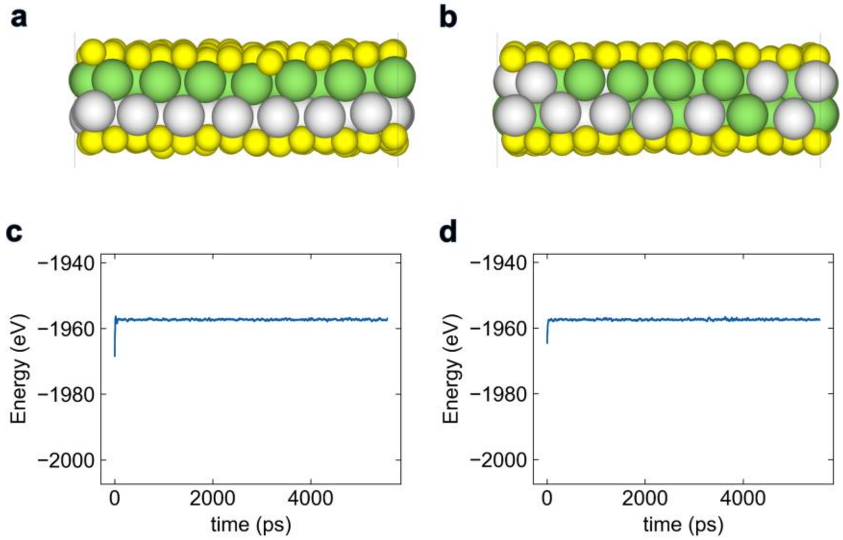

FIG. S9: Stability analysis of SMMS intermediate structures. (a-b) Atomic configurations of (a) SMoWS, and (b) alloyed $S(Mo_{0.5}W_{0.5})(W_{0.5}Mo_{0.5})S$ structures. (c-d) Corresponding energy evolution during 5500 ps MLP-MD simulations at 1100 K for (c) SMoWS, and (d) alloyed $S(Mo_{0.5}W_{0.5})(W_{0.5}Mo_{0.5})S$ structures, demonstrating their long-term stability. Note: The data has been smoothed using a low-pass filter for clarity.

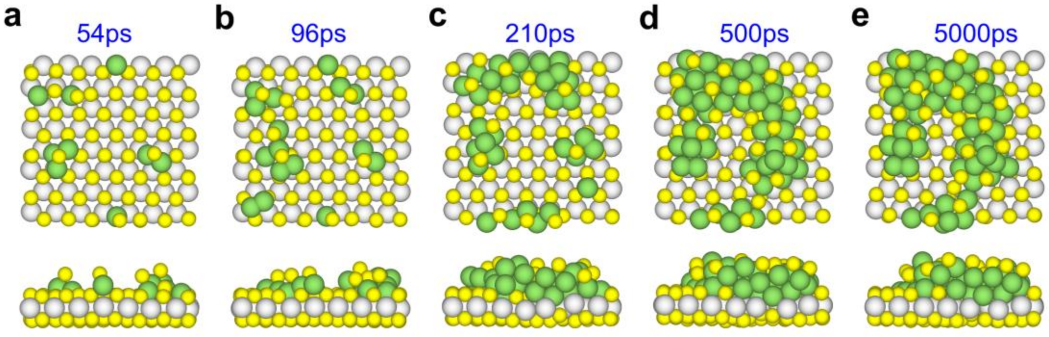

FIG. S10: (a-e) Snapshots of the growth structure of MoS_2/WS_2 vdWHs during the two-step vapor-deposition process (Mo atoms are deposited on WS_2) at 300 K.

4. SMMS Intermediate Structures to Bilayer TMDs

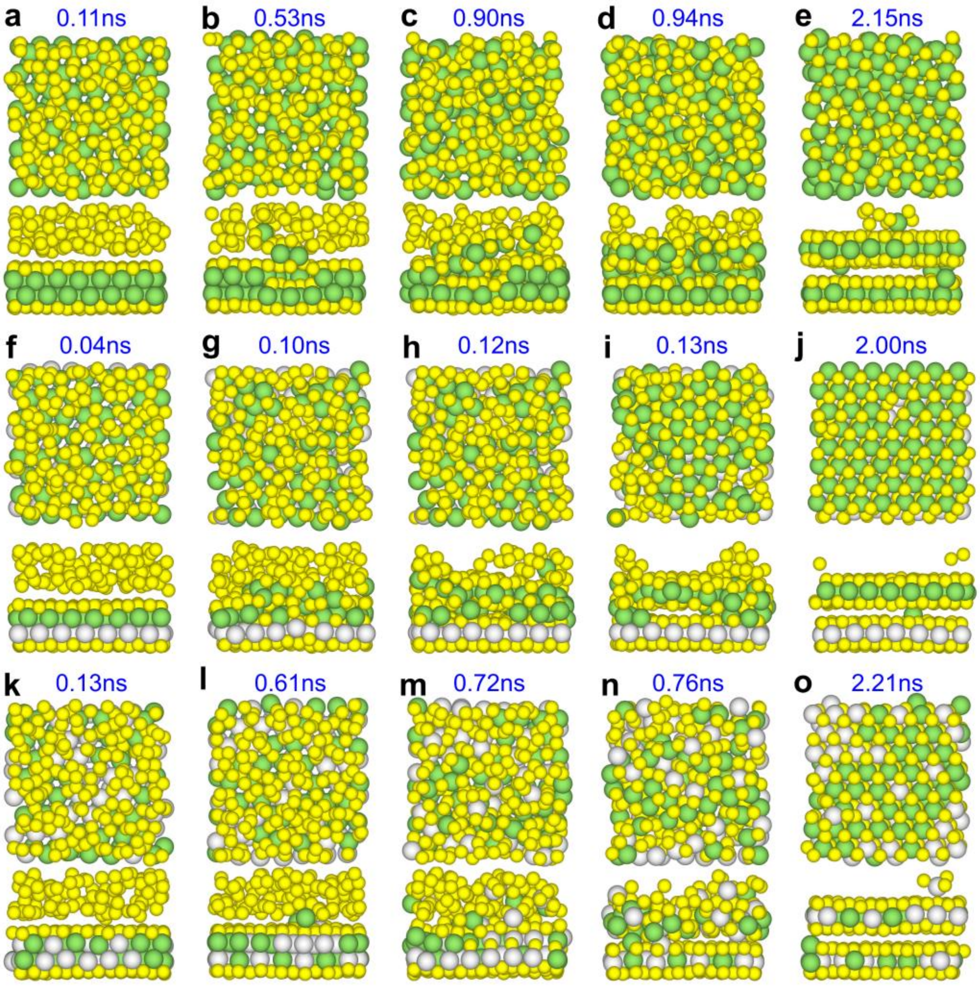

FIG. S11: (a-e) MLP-MD simulation with a large number of S atoms placed on top of the SMoMoS structure. The S atoms pull out Mo atoms, forming a bilayer MoS_2 structure (1100 K). (f-j) MLP-MD simulation with a large number of S atoms placed on top of the alloyed SMoWS structure. The S atoms pull out Mo atoms, forming a MoS_2/WS_2 vdWHs structure (1100 K). (k-o) MLP-MD simulation with a large number of S atoms placed on top of the alloyed $S(Mo_{0.5}W_{0.5})(W_{0.5}Mo_{0.5})S$ structure. The S atoms pull out Mo/W atoms, forming a bilayer alloyed $Mo_{0.5}W_{0.5}S_2$ structure (1100 K).

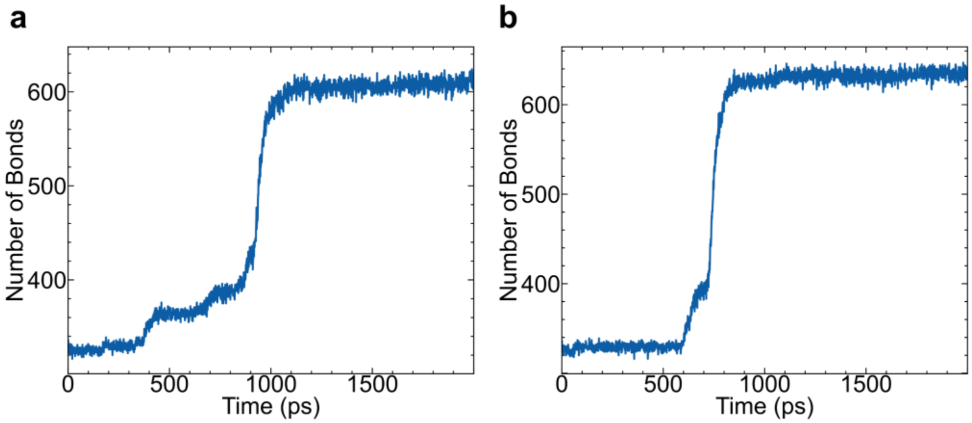

FIG. S12: (a) Evolution of the number of Mo-S bonds during sulfur deposition on the SMoMoS intermediate structure. Excess sulfur atoms extract Mo atoms from the SMoMoS structure, leading to the formation of a bilayer MoS_2 heterostructure. (b) Evolution of the number of Mo/W-S bonds during sulfur deposition on the alloyed $S(Mo_{0.5}W_{0.5})(W_{0.5}Mo_{0.5})S$ structure. Sulfur atoms facilitate simultaneous extraction of both Mo and W atoms, ultimately forming an alloyed bilayer $Mo_{0.5}W_{0.5}S_2$ configuration. A cutoff distance of 2.6 Å was applied for both Mo-S and W-S bond identifications. The bond counts include both pre-existing and newly formed bonds in the system. The initial number of bonds reflects the presence of metal–sulfur coordination in the intermediate structures, and as the bilayer TMD forms, the number of Mo–S bonds nearly double—indicating the completion of coordination environments in the emerging bilayer structure.

5. Simulation of MOCVD TMDs Growth

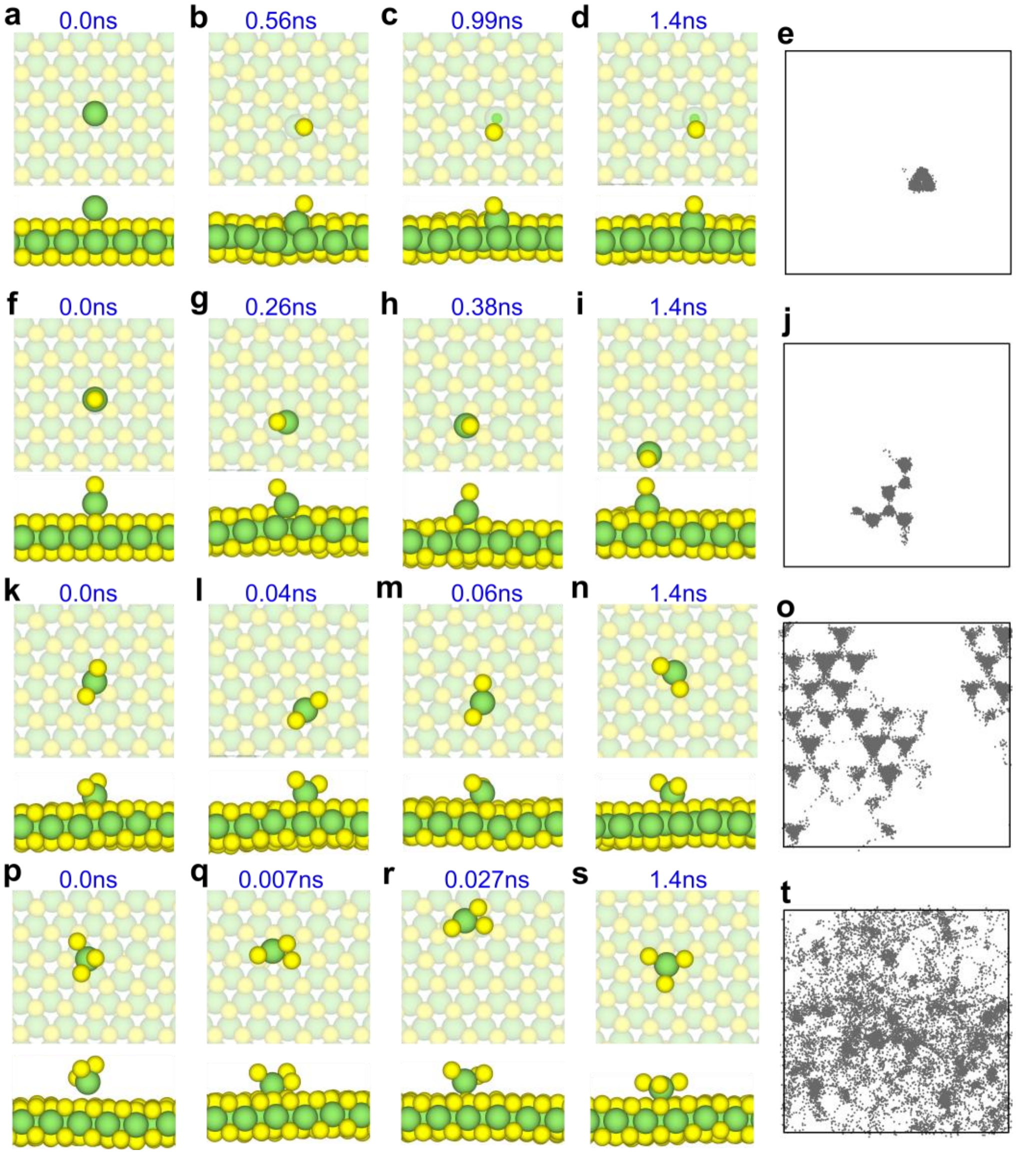

FIG. S13: MLP-MD simulations of various Mo-based clusters on MoS_2 (1100 K): (a-d) Single Mo atom deposition; (e) Mo atom trajectory on the ***xy*** plane; (f-j) Mo-S cluster; (k-o) Mo-S_2 cluster; (p-t) Mo-S_3 cluster.

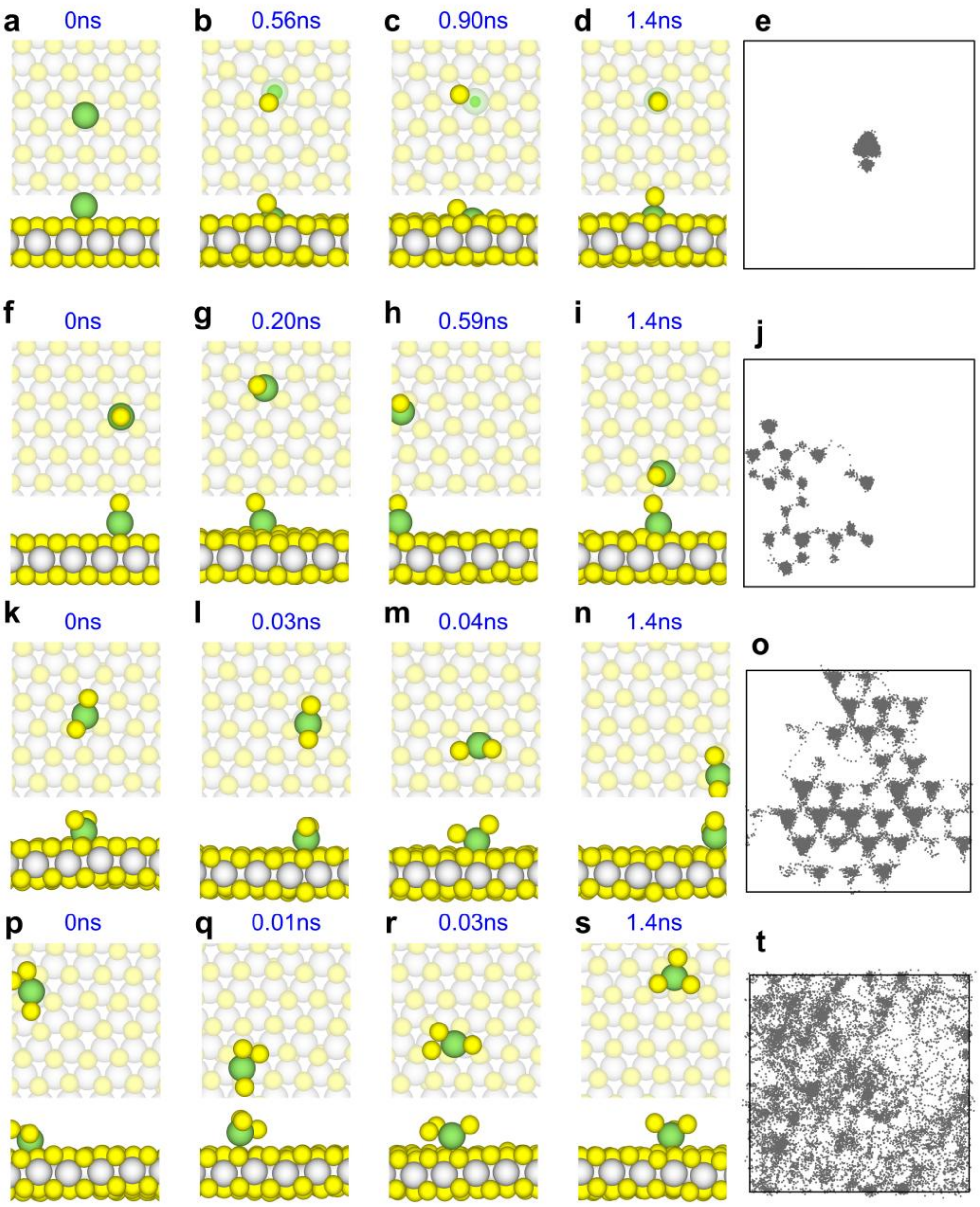

FIG. S14: MLP-MD simulations of various Mo-based clusters on WS_2 (1100 K): (a-d) Single Mo atom deposition; (e) Mo atom trajectory on the ***xy*** plane; (f-j) Mo-S cluster; (k-o) Mo-S_2 cluster; (p-t) Mo-S_3 cluster.

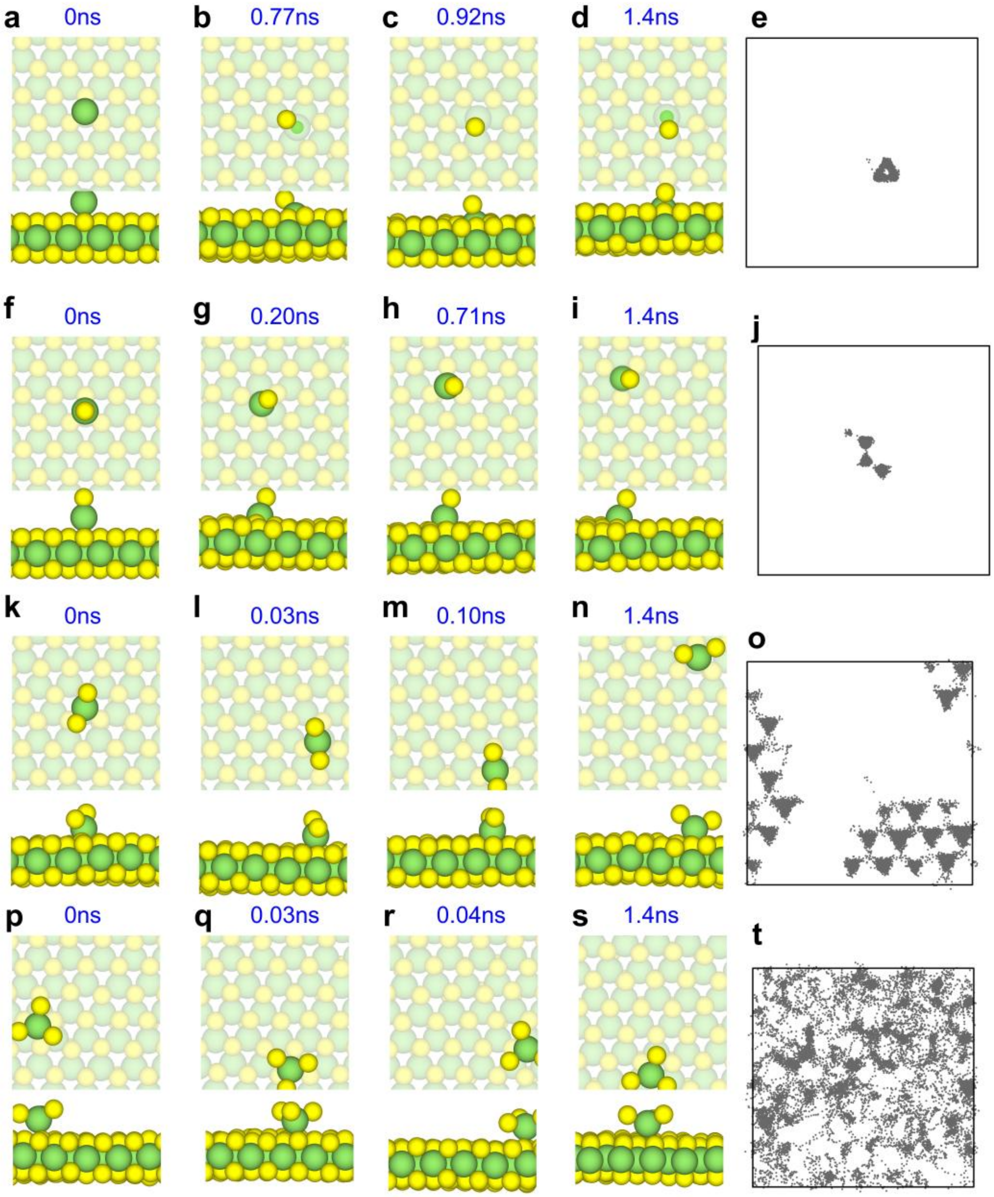

FIG. S15: MLP-MD simulations of various Mo-based clusters on MoS_2 (900 K): (a-d) Single Mo atom deposition; (e) Mo atom trajectory on the ***xy*** plane; (f-j) Mo-S cluster; (k-o) Mo-S_2 cluster; (p-t) Mo-S_3 cluster.

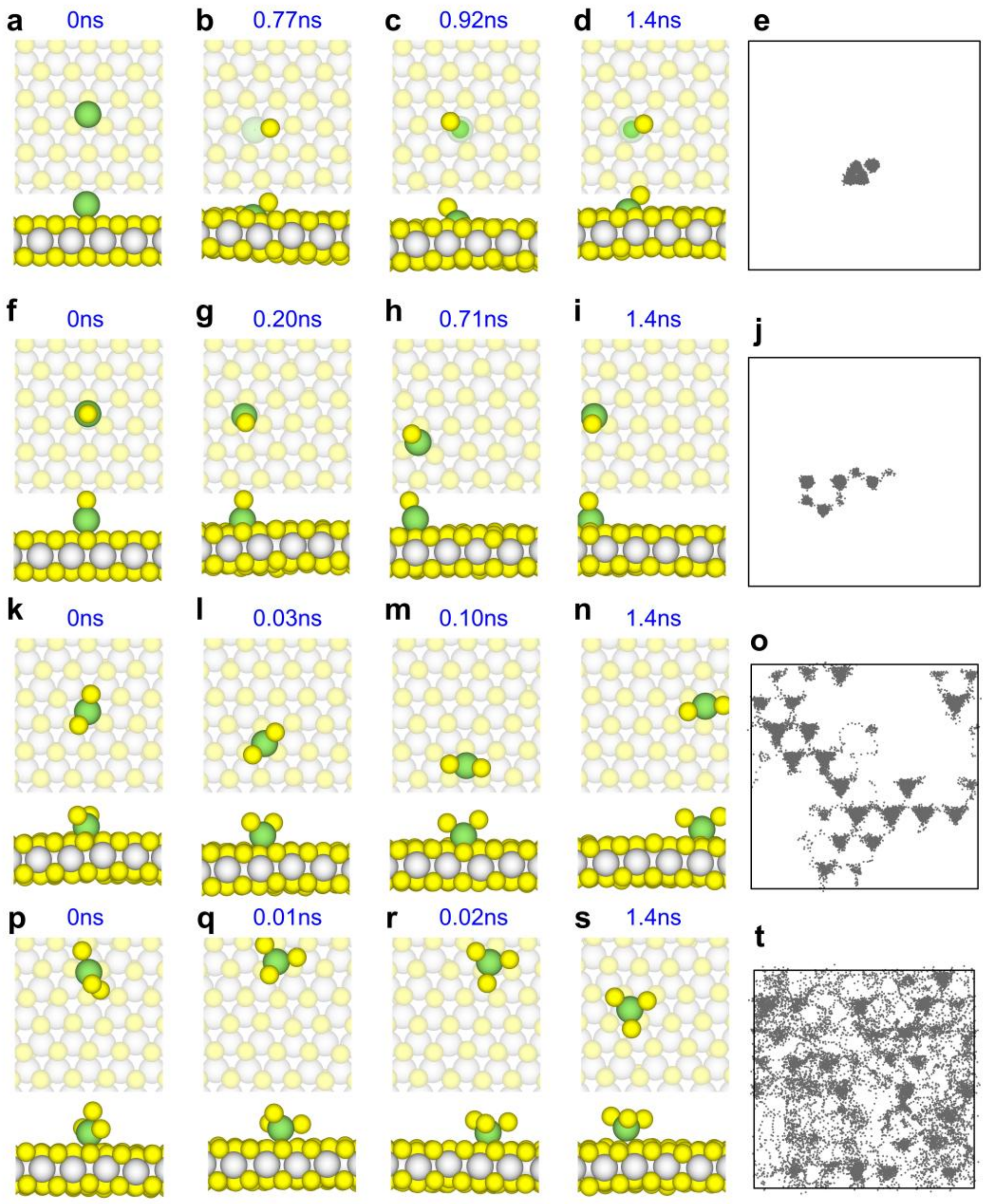

FIG. S16: MLP-MD simulations of various Mo-based clusters on WS_2 (900 K): (a-d) Single Mo atom deposition; (e) Mo atom trajectory on the ***xy*** plane; (f-j) Mo-S cluster; (k-o) Mo-S_2 cluster; (p-t) Mo-S_3 cluster.

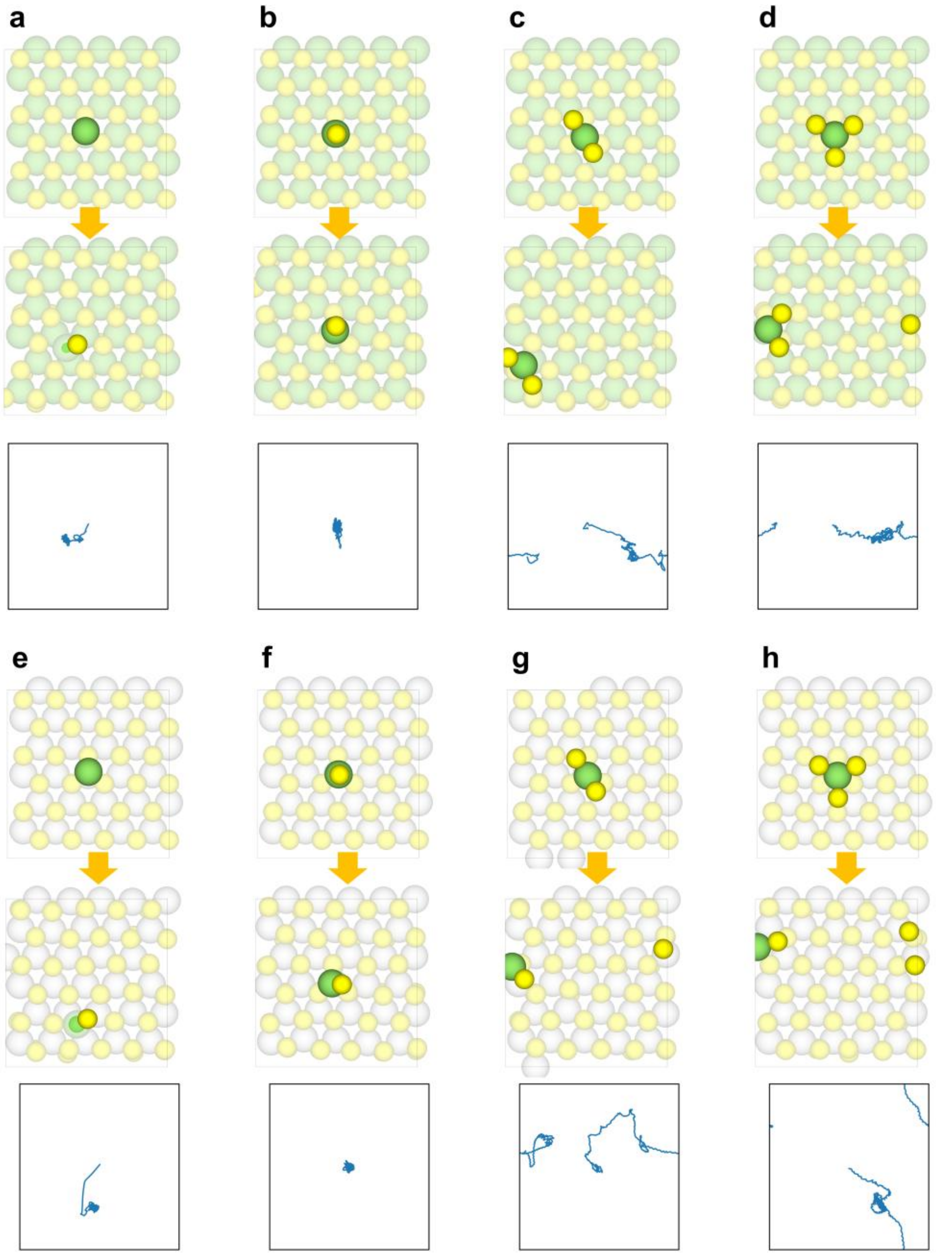

FIG. S17: Dynamical behaviour and migration trajectories of Mo-based clusters on monolayer MoS_2 and WS_2 surfaces (AIMD, 1100 K, 10 ps). Panels (a-d) show the structural evolution of Mo clusters on a MoS_2 surface, while (e-h) display the corresponding behaviour on a WS_2 surface. Each set of images includes the initial configuration, final configuration, and the in-plane (***xy***) trajectory of Mo atoms. All simulations were performed using AIMD based on the PBE functional under the NVT ensemble at 1100 K for a total duration of 10 ps.

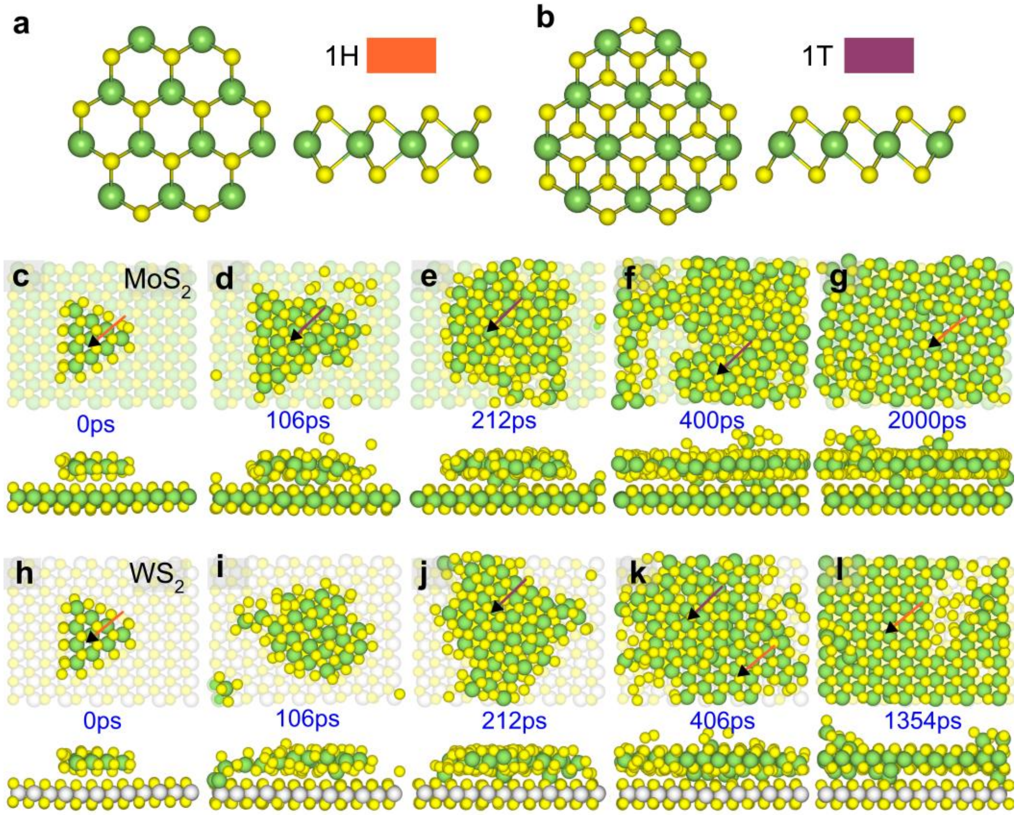

FIG. S18: (a-b) Schematic diagrams of (a) 1H-MoS_2 and (b) 1T-MoS_2 structures. MD simulations illustrating the growth processes of the second MoS_2 layer on different substrates: (c-g) MoS_2 monolayer substrate and (h-l) WS_2 monolayer substrate. The newly formed 1H-phase and 1T-phase domains during growth are indicated by orange and purple arrows, respectively (1100K).

6. Electronic Properties of the SMMS Contact with MoS_2

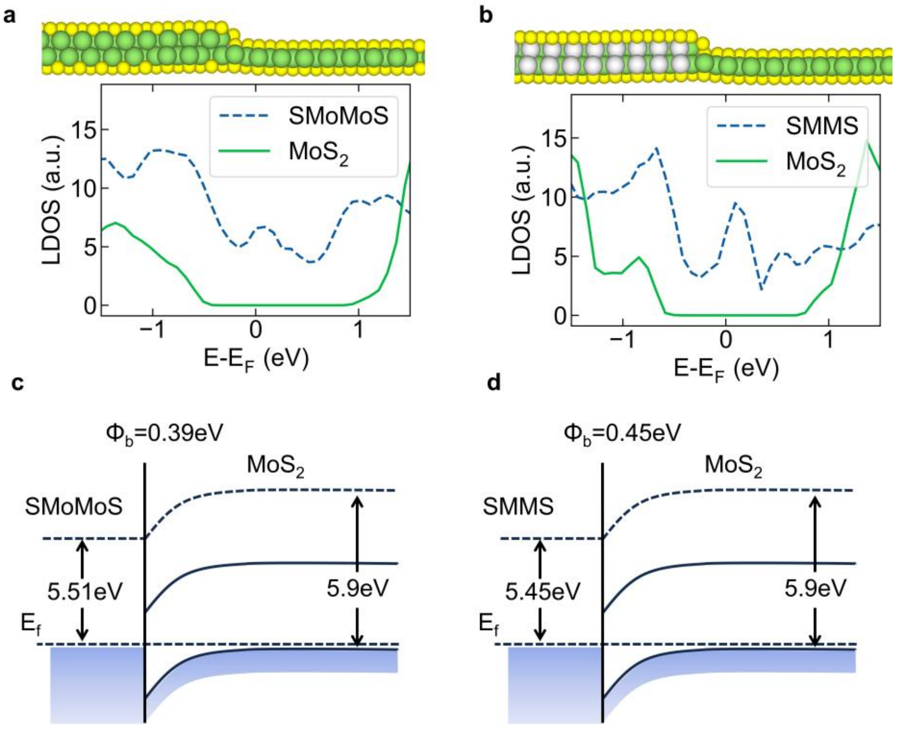

FIG. S19: Interfacial electronic properties of TMD heterostructures calculated using PBE functional. LDOS analysis at the interfaces: (a) SMoMoS-MoS_2 interface (SBH≈0.5 eV) and (b) alloyed SMMS-MoS_2 interface (SBH≈0.6 eV). Upper panels display atomic configurations (S: yellow, Mo: green, W: white). Lower panels show LDOS comparison between intermediate structures (SMoMoS/SMMS: blue dashed lines) and MoS_2 (green solid lines), with Fermi level aligned at 0 eV. Schottky-Mott limit analysis for p-type contacts: (c) SMoMoS-MoS_2 interface exhibits 0.39 eV SBH, (d) SMMS-MoS_2 interface exhibits 0.45 eV SBH.

To investigate the electronic properties and potential applications of the identified intermediate structures, we constructed heterostructures and calculated the p-type Schottky barrier height (SBH) using the PBE functional [2].

FIG. S19(a) shows the LDOS of the SMoMoS-MoS_2 interface, while FIG. S19(b) displays the LDOS of the alloyed SMMS-MoS_2 interface. The top panels illustrate the atomic configurations of these interfaces, with yellow, green, and white spheres representing S, Mo, and W atoms, respectively. In the bottom panels, the calculated LDOS is presented, where blue dashed lines

represent the LDOS of the intermediate structures (SMoMoS or SMMS), and green solid lines represent the LDOS of MoS_2. The Fermi level is set to 0 eV.

Analysis of these LDOS profiles reveals the formation of p-type Schottky barriers at both interfaces. For the SMoMoS-MoS_2 interface (FIG. S19(a)), we estimate a SBH of approximately 0.5 eV. In contrast, the alloyed SMMS-MoS_2 interface (FIG. S19(b)) exhibits a slightly higher barrier of about 0.6 eV. These findings are significant as they suggest that these intermediate structures, particularly SMoMoS, could potentially serve as low-resistance contacts for MoS_2-based electronic devices. The relatively low SBHs indicate favourable charge transport characteristics, which could be advantageous for various applications in nanoelectronics and optoelectronics utilizing TMD heterostructures.

References

[1] J. D. Morrow, J. L. A. Gardner, and V. L. Deringer, How to Validate Machine-Learned Interatomic Potentials, The Journal of Chemical Physics 158, 121501 (2023).

[2] R. T. Tung, The Physics and Chemistry of the Schottky Barrier Height, Applied Physics Reviews 1, 011304 (2014).
