# Supplementary material for: Intermediates of forming transition metal dichalcogenide heterostructures revealed by machine learning simulations
Source: Nat Commun. 2026 Feb 23;17:3086. doi: 10.1038/s41467-026-69977-x (PMC13039184; doi:10.1038/s41467-026-69977-x)
Supplement: Supplementary file 4 — Description of Additional Supplementary Files [file 41467_2026_69977_MOESM4_ESM.pdf]

Description of Additional Supplementary Files

File Name: Supplementary Movie 1.mp4

Description: A mixture of Mo:W:S in a ratio of 1:1:4 is annealed from 1500 K to 900 K using MLP-MD simulations. During the annealing process, multilayer structures of alloyed $Mo_xW_{1-x}S_2$ in the H phase are formed.

File Name: Supplementary Movie 2.mp4

Description: MLP-MD simulation of Mo atoms being deposited one by one onto the MoS_2 surface from vacuum. All Mo atoms are embedded into the MoS_2 during the simulation.

File Name: Supplementary Movie 3.mp4

Description: MLP-MD simulation of Mo atoms being deposited one by one onto the WS_2 surface from vacuum. All Mo atoms are embedded into the WS_2 during the simulation.

File Name: Supplementary Movie 4.mp4

Description: MLP-MD simulation with a large number of S atoms placed on top of the SMoMoS structure. The S atoms pull out Mo atoms, forming a bilayer MoS_2 structure.

File Name: Supplementary Movie 5.mp4

Description: MLP-MD simulation with a large number of S atoms placed on top of the alloyed $S(Mo_{0.5}W_{0.5})(W_{0.5}Mo_{0.5})S$ structure. The S atoms pull out Mo/W atoms, forming a bilayer alloyed $Mo_{0.5}W_{0.5}S_2$ structure.

File Name: Supplementary Movie 6.mp4

Description: MLP-MD simulation of simultaneous deposition of Mo and S atoms onto the MoS_2 surface. Initially, a 1T-like MoS_2 structure forms, but it transitions entirely to a 1H phase MoS_2 structure, resulting in a bilayer MoS_2.

File Name: Supplementary Movie 7.mp4

Description: MLP-MD simulation of simultaneous deposition of Mo and S atoms onto the WS_2 surface. Initially, a 1T-like MoS_2 structure forms, but it transitions entirely to a 1H phase MoS_2, resulting in a bilayer non-alloyed MoS_2/WS_2 vdWH.
